# Impact of roasting conditions on physicochemical, taste, volatile, and odor-active compound profiles of *Coffea arabica* L. (cv. Yellow Bourbon) using electronic sensors and GC–MS-O using a multivariate approach

**DOI:** 10.1016/j.fochx.2024.101119

**Published:** 2024-01-04

**Authors:** Seong Jun Hong, Chang Guk Boo, Sojeong Yoon, Hyangyeon Jeong, Seong Min Jo, Moon Yeon Youn, Jae Kyeom Kim, Young Jun Kim, Eui-Cheol Shin

**Affiliations:** a Department of GreenBio Science/Food Science and Technology, Gyeongsang National University, Jinju 52725, Republic of Korea; b DONG SUH OIL & FATS CO., Changwon 51397, Republic of Korea; c Department of Food and Biotechnology, Korea University, Sejong 30019, Republic of Korea

**Keywords:** *Coffea arabica* L. (cv. Yellow Bourbon), Physicochemical profiles, E-tongue, E-nose, GC–MS-olfactometry

## Abstract

This study investigated the effects of roasting conditions on the physicochemical, taste, and volatile and odor-active compound (OAC) profiles of *Coffea arabica* L. At 150 ℃, roasting increased chlorogenic acid, total flavonoids, and caffeine concentrations. However, umami and sourness sensor decreased during the roasting process. At 210 ℃ roasting, total flavonoid and caffeine concentrations increased during the roasting process. Aldehydes, ketones, and sulfur-containing compounds dramatically increased during the roasting at 210 ℃ for 20 and 30 min in E-nose analysis. Pyrazines were mainly generated during the roasting at 210 ℃ for 20 and 30 min, and pyrazines showed the highest concentrations among all OACs in GC-olfactometry (GC-O) analysis. E-tongue data showed the separation of beans by roasting temperature. However, the E-nose and GC-O data showed the separation of beans by both roasting temperature and time via multivariate analysis. We identified similar results and patterns in the E-nose and GC-O analyses.

## Highlights

• Coffea arabica L. (cv. Yellow Bourbon)’s taste and volatile profiles were analyzed using E-tongue and E-nose, and odor-active compounds were analyzed using GC–MS-O.
• Roasting process at 210℃ for 20 and 30 min changed higher differences in the number of odor-active compounds, taste, and volatile profiles compared to the unroasted bean.
• Taste and volatile patterns in *Coffea arabica L. (cv. Yellow Bourbon)* by the impact of roasting conditions were pointed out via multivariate analysis.
• The similar patterns between E-nose and GC-O data were identified via multivariate analysis.

## Introduction

Coffee is one of the most widely consumed drinks and is usually made using various brewing methods, including hot and cold brewing (Villanueva et al., 2006). The three main species of coffee are Arabica (*Coffea arabica*), Robusta (*Coffea canephora*), and Liberia (*Coffea liberica*) (Kim et al., 2007). Among these coffee species, Arabica accounts for approximately 70 % of total coffee production worldwide and is mainly produced in Latin America, Africa, and India (Mussatto et al., 2011, Ramos et al., 2016). The coffee market is steadily increasing annually, and owing to the coronavirus disease (COVID-19) pandemic, the number of people who want to enjoy coffee at home is increasing. The market for imported coffee machines in the Republic of Korea has increased approximately five-fold compared to that in 2010, and the market for imported coffee capsules and beans has increased approximately three-fold (Song, 2020, Yoon et al., 2022).

Coffee quality is influenced by several factors, such as green bean quality, coffee roaster type, roasting conditions, and the brewing process. Our previous studies have reported that the taste and odor characteristics of *Coffea arabica* L. (cv. Yellow Bourbon) showed different patterns according to different brewing methods (Jeong, Yoon, Jo, Hong, Kim, Kim, & Shin, 2023) and roaster types (Yoon et al., 2022). The roasting conditions are highly affected by the roasting temperature and time. During the roasting process, caramelization, the Maillard reaction, and other chemical reactions, including the degradation of amino acids, fatty acids, sugars, and chlorogenic acid, usually occur (Hyong et al., 2021, Yang et al., 2016). Therefore, dramatic changes in volatile compounds and taste quality can occur during and/or after roasting (Caporaso, Whitworth, Cui, & Fisk, 2018). Pyrazines, furans, esters, aldehydes, alcohols, pyridines, and nitrogen-containing volatiles are generated in coffee during roasting (Caporaso et al., 2018, Yang et al., 2016). These volatiles contribute to the characteristics of coffee flavors, such as roasted, caramel, nutty, sweet, and smoky odors (Kwon, Ahn, & Lee, 2021), which are well-known coffee aromas (Lee et al., 2020, Hong et al., 2020).

Coffee volatiles are generally measured using gas chromatography-mass spectrometry (GC–MS) and GC-olfactometry (GC-O) (Caporaso et al., 2018, Lee et al., 2020, Hong et al., 2020). Currently, various chemosensory instruments are increasingly being used to analyze flavor characteristics, including taste and volatile qualities (Yoon et al., 2022). The electronic tongue (E-tongue) and electronic nose (E-nose) are representative chemosensory instruments that are powerful analytical tools that are non-destructive, rapid, low-cost, and objective (Yoon et al., 2022). Additionally, the E-tongue and E-nose are highly similar to human sensory analysis because chemosensory instruments are based on the principles of mammalian taste and olfaction recognition (Yin et al., 2021). Although chemosensory instruments have some advantages, the E-nose system has a relatively lower identification quality than that of GC–MS. Therefore, combined approaches of GC–MS, E-nose, and E-tongue analysis of coffee samples are now being performed. Recent studies have measured the volatile profiles and taste properties of roasted coffee beans affected by different drying methods using E-tongue, E-nose, and GC–MS (Dong et al., 2019), and changes in the flavor profiles of Harbin red sausages affected by different roasting conditions were measured using GC–MS combined with electronic tongue and nose (Yin et al., 2021). Additionally, the flavor properties of cooked chicken drumsticks depending on sugar smoking times were measured using E-tongue, E-nose, and GC–MS (Zhang, Hu, Wang, Kong, & Chen, 2021).

Yellow Bourbon is an Arabica genotype with distinct flavor characteristics (Ramos et al., 2016). Despite several studies on coffee, research on Brazilian *Coffea arabica* L. (cv. Yellow Bourbon) is insufficient, as are roasted coffee beans, which can easily be utilized at home. Accordingly, the current study aimed to assess the impact of roasting conditions on the physicochemical profiles (DPPH, total flavonoid compounds, caffeine, and chlorogenic acid) and flavor characteristics (taste and volatile profiles) of *Coffea arabica* L. (cv. Yellow Bourbon) roasted at different temperatures for different times. In addition, the patterns of the obtained data were identified using multivariate analysis. Therefore, this study aimed to explore the impact of roasting conditions on the physicochemical, tasting, volatile, and odor-active characteristics of *Coffea arabica* L. (cv. Yellow Bourbon). Furthermore, the relationship between E-nose data and odor-active compound profiles (GC–MS-O data) was explored.

## Methods and materials

### Materials

NY2 FC grade *Coffea arabica* L. (cv. Yellow Bourbon), produced in Cerrado, Minas Gerais, Brazil, in 2021, was purchased from GSC International (Seoul, Republic of Korea).

### Roasting conditions

A coffee bean (15 g) was roasted under different conditions at 150 ℃ and 210 ℃ for 10, 20, and 30 min, respectively. Coffee roasting was performed using a coffee roaster (CBR-101A; GeneCafe Co., Ansan, Republic of Korea). Unroasted and roasted beans were ground to medium particle size (600 µm) using the coffee grinder (KG79, Delonghi Co., Treviso, Italy). Coffee samples were brewed with ground coffee bean (15 g) and 200 mL of water (95 °C) for 5 min using a hot brewing machine (CM151GKR, Tefal Co., Rumilly, Haute-Savoie, France) (Yoon et al., 2022, Jeong et al., 2023).

### Investigation of physicochemical profiles

Caffeine and chlorogenic acid concentrations were simultaneously analyzed using high-performance liquid chromatography (HPLC system; Agilent Technologies, Santa Clara, CA, USA). The coffee sample (1 mL) was filtered through a 0.45 μm filter, and the filtered extract (0.1 mL) was diluted with deionized water (0.9 mL). Under analysis conditions, the UV-detector (278 nm) and ZORBAX Eclipse XDB-C18 column (150 mm × 4.6 nm, 5 μm, YMC-Korea, Seongnam, Republic of Korea) were used. For the HPLC analysis conditions, the column temperature was at 25 °C, and the mobile phase (1 mL/min) was made by mixing distilled water (800 mL), acetic acid (1 mL), and methanol (200 mL) solutions. Standards (caffeine and chlorogenic acid) were purchased from Sigma-Aldrich (St. Louis, MO, USA) and diluted to 100 μg/g. The standard solution was prepared by mixing caffeine and chlorogenic acid. The injection volume was 20 μL, a calibration curve was prepared, and caffeine and chlorogenic acid concentrations in samples were calculated (Yoon et al., 2022).

The TFC was performed by diluting the samples to 1 mg/mL with distilled water. Diluted coffee samples (0.5 mL) were mixed with 95 % ethanol (1.5 mL), 1 M potassium acetate (0.1 mL), and 10 % aluminum nitrate (2.8 mL). After mixing, distilled water (2.8 mL) was added, and the mixture was allowed to react at room temperature for 30 min. The TFC was calculated using a standard material (quercetin), and pretreatment was performed in the same manner as the sample. Additionally, the calculation of TFC used the calibration curve (0.01, 0.02, 0.04, 0.06, and 0.08 g/ mL) obtained after absorbance measurement at 415 nm (Hong et al., 2020, Lee et al., 2020).

DPPH (1,1-diphenyl-2-picrylhydrazyl) radical scavenging activity was measured to determine the antioxidant capacity of the coffee samples. The samples were subjected to a series of dilutions in distilled water (0.1, 1, and 10 mg/mL). Next, a diluted sample (10 µL) was taken and mixed with 1 mL of 0.1 mM DPPH (dissolved in 99 % ethanol, Sigma-Aldrich Co., St. Louis, MO, USA), followed by incubation in the dark at 37 ℃ for 30 min. Absorbance was measured at 517 nm, and radical scavenging activity was calculated using Equation (1) (Lee et al., 2020, Hong et al., 2020).

(1) DPPHradicalscavengingactivity(%)=(1-Sampleabsorbance/blankabsorbance)×100

### Electronic tongue analysis

An e-tongue (ASTREE II, Alpha MOS Co., Toulouse, France) was used to measure the taste of coffee samples. Five basic taste sensors (sourness, SRS; saltiness, STS; umami, UMS; sweetness, SWS; and bitterness, BRS) were attached. To measure the components of the coffee sample, it (100 mL) was mixed with coffee extract (10 mL) and purified water (90 mL). The mixture for analysis was placed in contact with the e-tongue sensor for 2 min and each taste sensor value was measured via sample contact. The sensors were washed with purified water to reduce errors caused by cross-contamination between samples during the analysis (Yoon et al., 2022).

### Electronic nose analysis

An E-nose (HERACLES Neo, Alpha Mos Co., Toulouse, France) was used to measure the volatile compounds in the coffee samples. The coffee extract was put (2 mL) in the headspace vial (22.5 × 75 mm, PTFE (polytetrafluoroethylene)/silicone septum, aluminum cap), and volatiles in the headspace of the vial were collected during stirring (30 G) at 50 °C for 20 min using an E-nose incubator. After the volatiles collection, volatiles (5000 μL) were collected by an automatic sampler attached to the E-nose. MXT-5 column (2 m × 0.18 mm, 5 % diphenyl / 95 % dimethyl polysiloxane, Alpha MOS Co., Toulouse, France) and flame ionization detector (FID; Alpha MOS Co., Toulouse, France) were used and attached to the E-nose. The FID temperature was set at 260 °C, and the hydrogen flow rate was set to 1 mL/min. During volatiles acquisition, the acquisition time was 277 s for each sample, and trap absorption and desorption temperatures were 40 ℃ and 250 ℃, respectively. The oven temperature was maintained at 40 °C for 5 s, increased to 270 °C at a rate of 4 °C/s, and maintained for 30 s. In qualitative analysis, 20 uL of headspace injection solution (C_6_-C_16_) was injected into the E-nose. Each volatile compound was identified using Kovat’s index (the retention index in terms of carbon number) library-based AcroChembase (Alpha Mos Co.), and the volatile compounds corresponding to each peak were measured (Yoon et al., 2022).

### GC–MS-O analysis

Odor-active compounds (OACs) in coffee extracts were measured using GC–MS-O, and OACs were collected using headspace solid-phase microextraction (HS-SPME; 50/30 μm divinylbenzene/carboxen/polydimethylsiloxane). The coffee sample (5 mL) was put in the headspace vial, sealed with an aluminum cap, and exposed to the headspace of the heated sample at 50 ℃ for 20 min (equilibrium). The absorption time of OACs was 30 min using a solid-phase microextraction (SPME) fiber, and the desorption time was 25 min. The total analysis time was 46 min, and the OACs analysis time was 20 min (5–25 min) using GC–MS (Agilent 7890A & 5975C, Agilent Technologies, Santa Clara, CA, USA) coupled with GC-O (olfactory detection port-3 (ODP-3), Gerstel Co., Linthicum, MD, USA). The OACs intensity was expressed on four levels, ranging from 1 to 4, with a higher number representing a higher odor activation level. In addition, the odor descriptions of the OACs were identified using GC-O. The analysis column was the HP-5MS column (30 m × 0.25 mm i.d. × 0.25 μm film thickness). The oven temperature was at 40 ℃ for 5 min and accelerated to 200 ℃ at a rate of 5 ℃/ h. The injector temperature was 220 ℃, and the helium (He) flow rate was 1.0 mL/min (splitless mode). A total ionization chromatogram (TIC) was used to separate the peaks, and the mass spectrum library (NIST 12) and literature were used to identify the volatile flavor compounds. The concentration of OAC was calculated by converting the peak area of an internal standard (pentadecane; 0.005 μg), and each OAC was expressed as μg/g. The retention index (RI) was calculated for the qualitative analysis.

RI was determined by the following Equation (1).

(1) RIx=100n+100-((tRx-tRn)/(tRn+1-tRn))

where RIx is the RI of the unknown compound, tRx is the retention time of the unknown compound, tRn is the retention time of the *n*-alkane, and tRn + 1 is the retention time of the next *n*-alkane. tRx lies between tRn and tRn + 1 (n = number of carbon atoms) (Hong et al., 2021).

### Statistical analysis

This study was performed six times (E-tongue), in triplicate (physicochemical profiles and E-nose), and duplicate (GC–MS-O), and the results were expressed as mean ± SD. Statistical significance was confirmed when the *p*-value was < 0.05, and Tukey’s multiple range test was used to compare means using SAS (Statistical Analysis System, version 9.0; SAS Institute Inc., Cary, NC, USA). In addition, the tastes, volatiles, and OACs patterns of all samples were identified via multivariate analyses, including principal component analysis (PCA) and cluster analysis (CA). Multivariate analyses were performed for taste, volatile, and odor-active compound datasets using the XLSTAT statistics software ver. 9.2 (Addinsoft, New York, NY, USA) (Hong et al., 2021).

## Results and discussion

### Caffeine and chlorogenic acid

The validation results for caffeine and chlorogenic acid are presented in Table 1. The concentrations of caffeine and chlorogenic acid were measured in unroasted and roasted bean extracts, and the results are shown in Table 2. Caffeine concentrations significantly increased after 20 min (20 and 30 min) (*p* < 0.05) at 150 ℃ roasting; however, the sample roasted for 10 min did not show any significant differences compared to unroasted bean extract (*p* > 0.05). Caffeine concentrations in roasted samples significantly increased compared to those of the unroasted bean at 210 ℃ roasting, regardless of roasting times (*p* < 0.05). Particularly, the sample roasted for 10 min (210 ℃) had the highest concentration among all samples (*p* < 0.05), and the concentrations of caffeine decreased in a time-dependent manner after the 210 ℃ roasting at 10 min (*p* < 0.05). Moreover, a roasted bean (210 ℃) at 20 min significantly decreased caffeine content compared to that of the unroasted bean (*p* < 0.05). In chlorogenic acid concentrations, most of the samples roasted at 150 ℃ significantly increased chlorogenic concentrations, except for a sample roasted at 10 min (*p* < 0.05). In particular, the sample roasted for 20 min showed the highest chlorogenic concentration among all the samples (*p* < 0.05). After a roasting temperature of 210 ℃, a sample roasted for 10 min increased chlorogenic concentration compared to an unroasted bean (*p* < 0.05); however, samples roasted for 20 and 30 min decreased chlorogenic concentrations in a time-dependent manner (*p* < 0.05).

**Table 1:** Method validation of caffeine, chlorogenic acid, and total flavonoid compounds.

	Calibration curve	r^2^	LOD^1)^ (mg/mL)	LOQ^2)^ (mg/mL)
Caffeine	Y = 0.017558 x + 0.097971	0.9999	0.015	0.045
Chlorogenic acid	Y = 0.228174 x + 0.827800	0.9955	0.005	0.014
TFC^3)^	Y = 0.097890 x - 0.006822	0.9995	2.500	7.500

^1)^LOD: limit of detection.

^2)^ LOQ: limit of quantitation.

^3)^ TFC: total flavonoid compounds.

**Table 2:** Physicochemical profiles in coffee extracts using the roasting processes.

	Roasting	Roasting time (min)			
	temperature (℃)	Unroasted	10 min	20 min	30 min
Caffeine (mg/mL)	150 ℃	1.75 ± 0.02^b1)^	1.71 ± 0.02^b^	1.84 ± 0.02^a^	1.84 ± 0.01^a^
Chlorogenic acid (mg/mL)		5.76 ± 0.06^c^	5.52 ± 0.09^d^	6.29 ± 0.09^a^	5.97 ± 0.02^b^
TFC^2)^ (mg/mL)		79.37 ± 0.12^c^	80.12 ± 0.20^ab^	79.84 ± 0.17^b^	80.49 ± 0.16^a^
DPPH radical scavenging activity(%)		32.85 ± 0.55	34.29 ± 1.56	35.61 ± 0.56	32.66 ± 1.67
Caffeine (mg/mL)	210 ℃	1.75 ± 0.02^c^	1.96 ± 0.01^a^	1.91 ± 0.01^b^	1.70 ± 0.01^d^
Chlorogenic acid (mg/mL)		5.76 ± 0.06^b^	6.56 ± 0.05^a^	5.42 ± 0.03^c^	4.10 ± 0.01^d^
TFC (mg/mL)		79.37 ± 0.12^c^	80.10 ± 0.12^b^	80.08 ± 0.06^b^	80.47 ± 0.05^a^
DPPH radical scavenging activity(%)		32.85 ± 0.55	34.19 ± 0.99	32.70 ± 1.12	32.86 ± 1.05

Data are given as mean ± SD values from experiments performed in triplicate.

^1)^ Mean values with different letters (a-d) within the same row are significantly different according to Tukey’s multiple range test (*p* < 0.05).

^2)^ TFC: total flavonoid compounds.

Caffeine is an alkaloid substance, and caffeine intake generally controls the central nervous system (Hong et al., 2020, Lee et al., 2020, Yoon et al., 2022). Caffeine has a higher thermostability than chlorogenic acid, and this study also identified a higher thermostability of caffeine, in agreement with previous findings (Mehaya & Mohammad, 2020). In addition, caffeine tastes bitter and exhibits relatively high stability under hot conditions (Yoon et al., 2022). Chlorogenic acid is a phenolic substance, and unroasted beans contain 4–6 % of this compound. Chlorogenic acid is usually decomposed during roasting and converted into secondary metabolites (quinic acid and caffeic acid), which contribute to the bitter taste of coffee (Yoon et al., 2022). Moreover, chlorogenic acid plays an important role in determining coffee quality (Kim & Park, 2006). In this study, caffeine content showed an increasing tendency after roasting processes, excluding the samples roasted at 150 ℃ for 10 min and 210 ℃ for 30 min. A previous study reported that roasted coffee beans (160, 180, and 220 ℃) increased caffeine content compared to unroasted beans (Mehaya & Mohammad, 2020). Meanwhile, chlorogenic acid content increased after roasting at 150 ℃ for 20 min, however, it decreased after 210 ℃ for 20 min. A previous study reported that chlorogenic acid contents in roasted fennel seed (≥ 150 ℃) significantly increased compared to that of unheated seed (Hayat, Abbas, Hussain, Shahzad, & Tahir, 2019). Previous studies have reported that roasted beans (lightly roasted) have a higher caffeine content than unroasted beans. Caffeine has high thermostability, and moisture decreases during roasting. Additionally, roasted coffee beans (less time) have higher denser caffeine (Hečimović et al., 2011, Mehaya and Mohammad, 2020, Hong et al., 2020, Lee et al., 2020). Therefore, most of the beans roasted in this study may have had a significantly higher caffeine content than unroasted beans. However, in this study, chlorogenic acid showed a faster decreasing tendency than caffeine. Accordingly, the thermostability of chlorogenic acid is relatively low during roasting. These results demonstrate that caffeine and chlorogenic acid contents changed with roasting temperature compared to changes in caffeine content. Moreover, the decomposition of caffeine was only shown at 210 ℃. However, the decomposition of chlorogenic acid was shown at 150 ℃ and 210 ℃.

### Total flavonoid compounds and DPPH radical scavenging

The validation of TFC is presented in Table 1. The concentrations of TFC and DPPH radical scavenging activity were measured in unroasted and roasted bean extracts, and the results are shown in Table 2. At 150 ℃, all roasted samples significantly increased TFC compared to that of unroasted beans (*p* < 0.05), and a sample roasted for 30 min had the highest TFC concentrations among all samples (*p* < 0.05). At 210 ℃, all roasted samples also significantly increased TFC compared to the unroasted bean (*p* < 0.05), similar to the samples roasted at 150 ℃. Additionally, a sample roasted for 30 min (210 ℃) had the highest TFC among all samples (*p* < 0.05). In DPPH radical scavenging (%), a few roasted samples showed an increasing tendency after the roasting processes, however, no significant differences were observed between extracts of unroasted bean and roasted bean (150 ℃ and 210 ℃) (*p* > 0.05).

TFC is a secondary metabolite in plants generated from chalcone, which is produced in response to malonyl-CoA and coumaroyl-CoA via the shikimate pathway. In addition, TFC has antioxidant and antiviral properties (Hong et al., 2020, Lee et al., 2020). DPPH radical scavenging is a measure of antioxidant capacity and is positively correlated with TFC content (Hong et al., 2020, Lee et al., 2020, Hayat et al., 2019). These indicators typically increase during or after roasting (Ahmed et al., 2021, Hayat et al., 2019, Hong et al., 2020, Lee et al., 2020). Previous research reported that roasted beans increased TFC (Hečimović et al., 2011). During roasting, the molecular bonds in the cell matrix are generally destroyed, the solubility of the molecules increases, and insoluble TFC is extracted (Hayat et al., 2019, Hong et al., 2020, Lee et al., 2020). Accordingly, it usually increases after roasting. In this study, TFC was significantly increased during roasting, except for 150 ℃ for 10 min (*p* < 0.05). Previous studies reported that DPPH in *Coffea Arabica* (Ethiopia) significantly decreased during the roasting at ≤ 160 ℃ (Mehaya & Mohammad, 2020), and DPPH and TFC in *Coffea canephora* (Robusta) significantly decreased during the roasting at ≤ 210℃ (Chindapan, Chaninkun, & Devahastin, 2022). In the present study, the TFC of *Coffea arabica L. (cv. Yellow Bourbon)* significantly increased (*p* < 0.05), while DPPH did not show any significant differences among the beans (*p* > 0.05). These results suggest that *Coffeea Arabica* L. (cv. Yellow Bourbon) may have a higher antioxidant thermostability than *Coffeea Arabica* (Ethiopia) and *Coffeea canepora* (Robusta).

### Electronic tongue

**Fig. 1:**
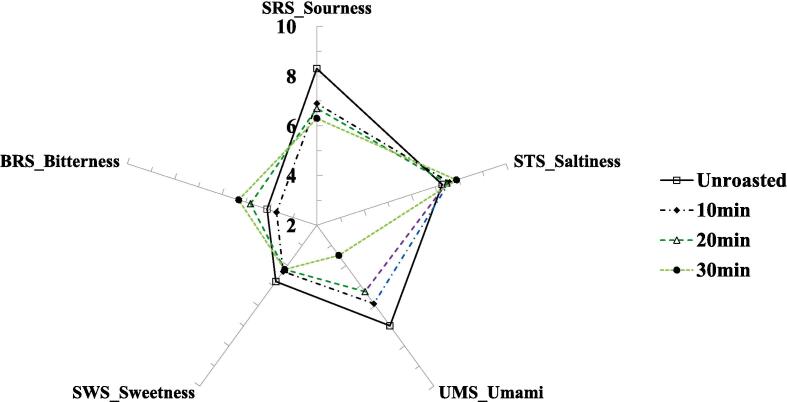

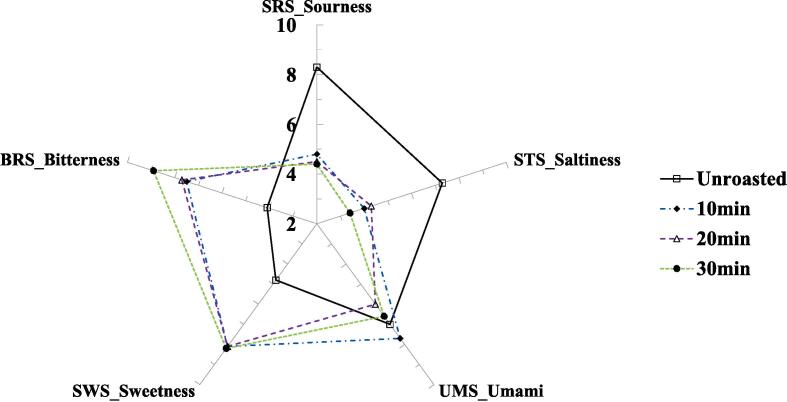

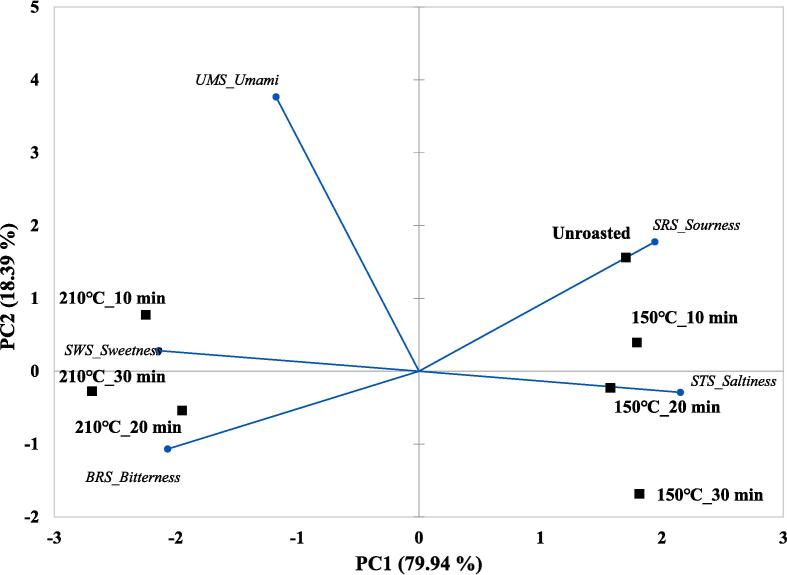

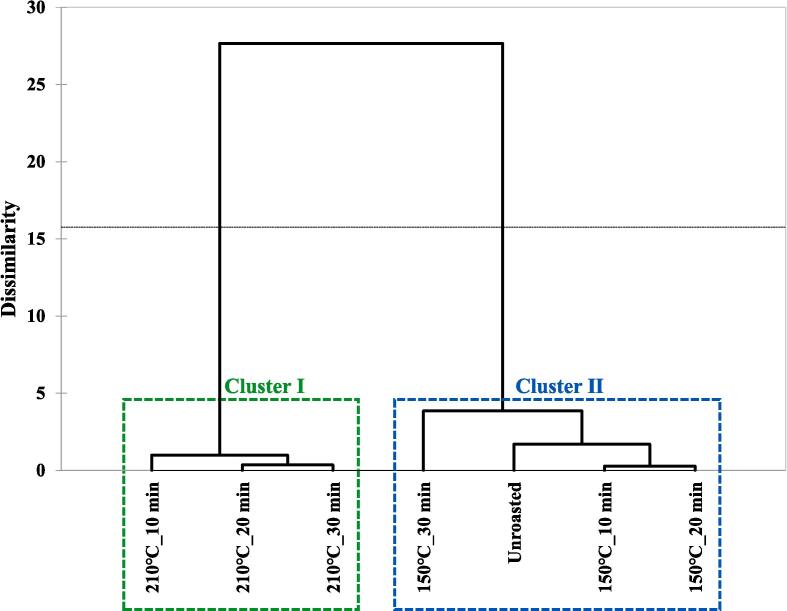
Radar plot for taste profiles in roasted coffee samples at 150 ℃ (A), 210 ℃ (B), PCA-biplot for taste dataset between unroasted- and roasted-coffee samples (C), dendrogram for taste dataset between unroasted and roasted coffee samples (D).

The taste of *Coffea arabica (cv. Yellow Bourbon) was measured* during the roasting process, and the results are shown in Fig. 1. At 150 ℃ roasting (Fig. 1A), the SRS of the unroasted bean showed the highest value among all samples, and SRS showed a decreasing tendency after roasting. The UMS of the unroasted samples showed the highest value among all the samples, and the UMS showed a decreasing tendency after roasting in a time-dependent manner. The sample roasted for 30 min exhibited the lowest UMS value. The BRS of most roasted beans showed an increasing tendency; however, fewer changes occurred compared to unroasted beans. Slight differences in the values between unroasted and roasted beans were observed for STS and SWS.

SRS and STS showed a decreasing tendency after the roasting processes at 210 ℃ (Fig. 1B). However, SWS and BRS were dramatically increased after the roasting processes, unlike roasting at 150 ℃. In particular, BRS increased in a time-dependent manner. UMS in the roasted bean (10 min) was increased, unlike roasting at 150 ℃. UMS in other roasted beans was decreased. Nevertheless, unroasted and roasted beans exhibited few differences, unlike the samples roasted at 150 ℃. Changes in roasted beans (210 ℃) in five taste sensor values showed a dynamic change compared to lower temperatures (150 ℃). In particular, BRS, SWS, and STS showed less change in roasted beans at 150 ℃ compared to the unroasted beans; however, these three taste values showed greater changes after the roasting processes at 210 ℃.

E-tongue is used to analyze the quality and taste characteristics of numerous foods (Hong et al., 2022). The e-tongue responds to the electrical signals of taste-related compounds to classify each nonvolatile compound in food (Hong et al., 2022). The e-tongue can discriminate between numerous food samples based on non-volatile compounds in food samples (Dong et al., 2017, Dong et al., 2019, Hong et al., 2021, Hong et al., 2022, Yin et al., 2021, Yoon et al., 2022). In this study, SRS decreased with roasting in a time-dependent manner, regardless of the roasting temperature, and SRS played an important role in discriminating between unroasted and roasted beans. Sour taste is considered an important factor in determining food quality, and roasting generally decreases the sour taste of coffee beans (Yoon et al., 2022). Additionally, these results may be related to a reduction in acidity and source-related amino acids (Lee et al., 2020, Hong et al., 2020, Yoon et al., 2022). The bitter taste is also important in coffee (Lee et al., 2020, Hong et al., 2020, Yoon et al., 2022), and the BRS increases during roasting. A previous study reported that the concentrations of caffeine and bitter-tasting amino acids were associated with the bitter taste of coffee (Lee et al., 2020, Hong et al., 2020), and another study reported that the decomposition of chlorogenic acid was also associated with the bitter taste of coffee due to the conversion of chlorogenic acid to quinic and caffeic acids (Yoon et al., 2022). In this study, caffeine increased during the roasting, regardless of roasting temperatures; however, chlorogenic acid only decreased during a higher temperature roasting (210 ℃). Accordingly, increased BRS may be related to the combined effects of changes in caffeine and chlorogenic acid concentrations during rotation. The roasted beans at 210 ℃ dramatically increased SWS compared to the unroasted beans, unlike the roasted beans at 150 ℃. A previous study reported that roasting usually converts macromolecules to smaller molecules, such as the decomposition of polysaccharides into monosaccharides (Kwon et al., 2021). In addition, roasting induces the Maillard reaction and caramelization (Kwon et al., 2021). Accordingly, the increased SWS may be related to the decomposition of sugars via the Maillard reaction and caramelization.

### Taste patterns in unroasted and roasted beans via multivariate analysis

Taste patterns in unroasted and roasted beans (150 and 210 ℃) were identified via multivariate analyses (PCA and CA) based on the changes in taste characteristics using E-tongue. The results are shown in Fig. 1C-D. The principal components (PCs) showed 98.33 % variance, including 79.94 % variance (PC1) and 18.39 % variance (PC2). Among all samples, roasted beans (210 ℃) and other samples were separated by the PC1 axis. Roasted beans (210 ℃) were located on the negative axis of PC1; however, roasted (150 ℃) and unroasted beans were located on the positive axis of PC1. A sample roasted at 210 ℃ for 10 min was mainly influenced by SWS, and other roasted samples (20 and 30 min) were mainly influenced by SWS and BRS. The unroasted bean was mainly influenced by SRS, and roasted beans at 150 ℃ were influenced by STS. Particularly, a sample roasted at 150 ℃ for 20 min was highly influenced by STS compared to other beans roasted at 150 ℃. Unlike the results for the PC1 axis, the samples were separated by the PC2 axis according to the roasting time. Both unroasted and roasted beans (10 min) were located on the positive axis of PC2; however, the roasted beans shifted downward. In roasted beans (150 ℃), they shifted downward in a time-dependent manner, and these samples showed higher separation compared with roasted beans (210 ℃). In the CA results (Fig. 1D), roasted beans (210 ℃) were classified as cluster I and unroasted and roasted beans (150 ℃) were classified as cluster II. In cluster II, unroasted and roasted beans (150 ℃) showed relatively lower dissimilarity compared to roasted beans (210 ℃). The CA results showed that the roasting temperatures could be discriminated against by the clusters. Recent studies have reported that differences in the taste patterns of coffee based on e-tongue analysis data were identified according to drying processing methods via chemometrics (Dong et al., 2019). In this study, taste patterns were identified mainly via chemometrics according to roasting temperature.

### Electronic nose

**Fig. 2:**
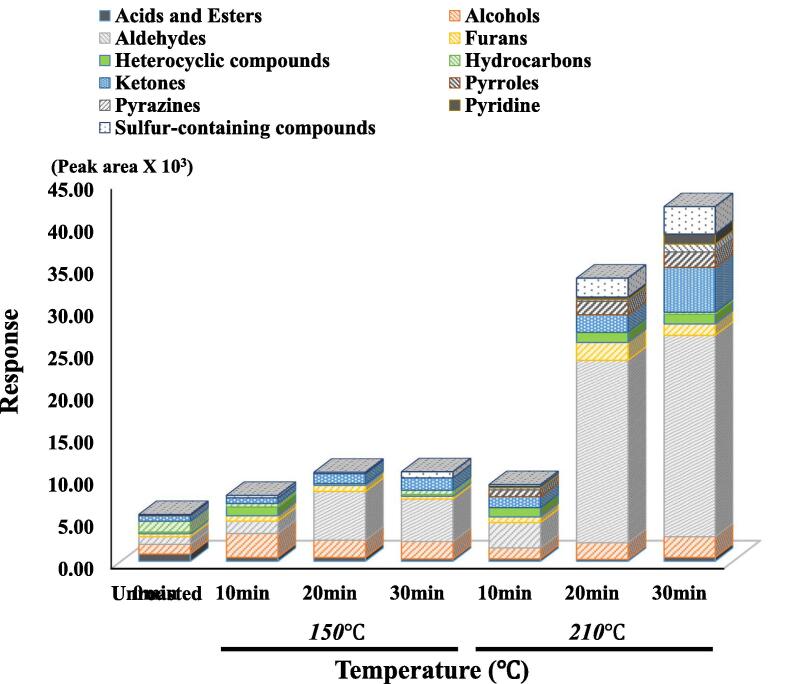

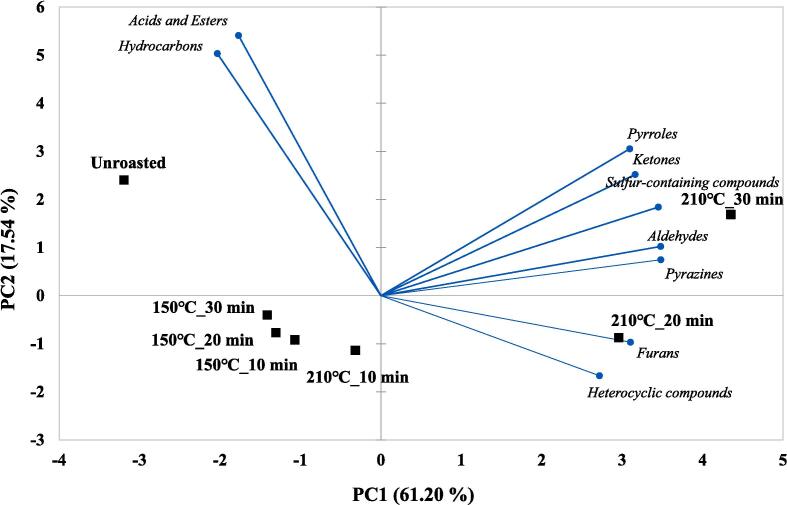

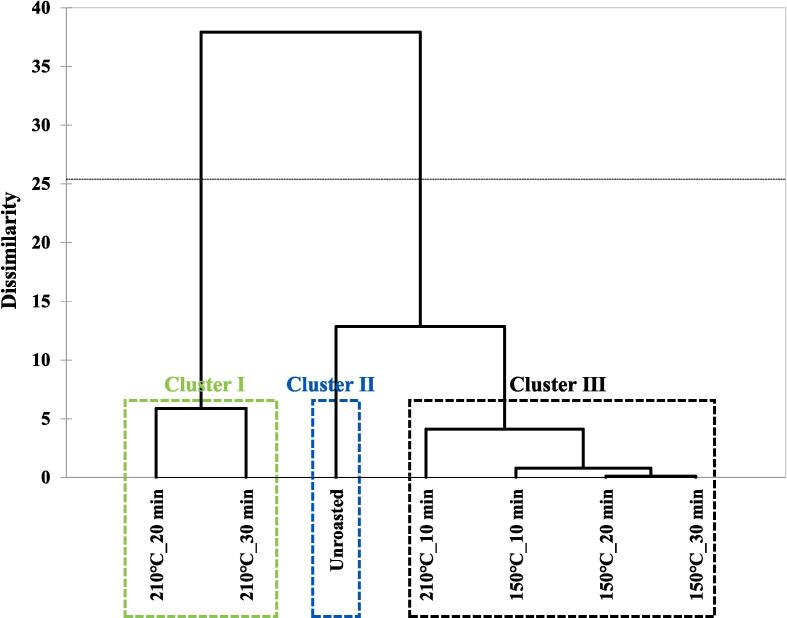

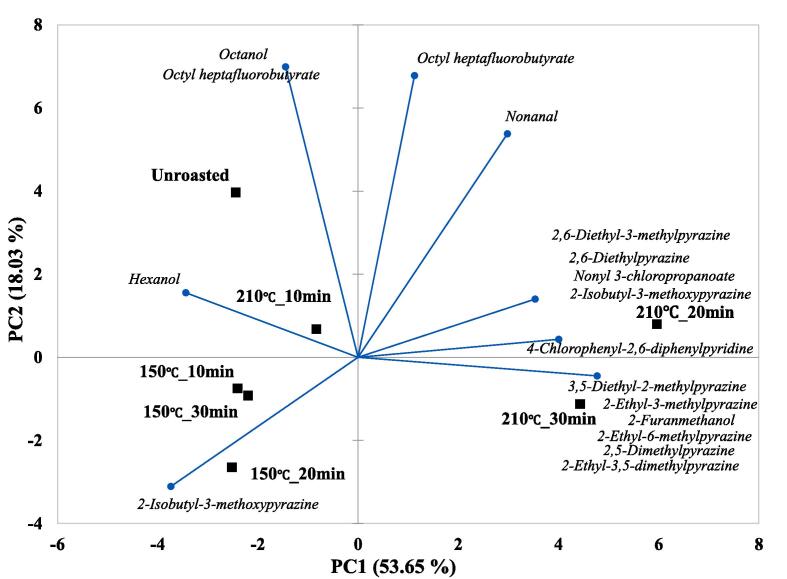
Volatile compound profiles between unroasted- and roasted-coffee samples using E-nose (A), PCA-biplot for volatiles dataset between unroasted- and roasted-coffee samples (B), dendrogram for volatiles dataset between unroasted- and roasted-coffee samples (C).

The volatile compounds in the unroasted and roasted beans were measured using an E-nose system, and the results are shown in Fig. 2A. The volatile compounds detected in this study include acids, esters, alcohols, aldehydes, furans, heterocyclics, hydrocarbons, ketones, pyrroles, pyrazines, pyridines, and sulfur-containing compounds. The roasting process induced an increasing tendency for volatile concentrations compared to unroasted beans. Particularly, higher temperature roasting (210 ℃) dramatically increased the total volatile concentrations after 20 min. The alcohols, aldehydes, ketones, and sulfur-containing compounds showed dynamic changes during roasting. The concentrations of alcohols increased during the roasting at 10 min (150 ℃), and the decomposition of alcohols was shown after 20 min. However, the concentrations of alcohols increased in a time-dependent manner during the roasting at 210 ℃. The concentration of aldehydes increased during the roasting process, regardless of the roasting temperature. In both the roasting at 150 ℃ and 210 ℃, aldehyde concentrations were increased by 20 min roasting compared to that of 10 min rosting. In roasting at 150 ℃, aldehyde concentrations decreased after 30 min, but aldehyde concentrations increased in a time-dependent manner during the roasting at 210 ℃. Particularly, the roasting processes at 210 ℃ for 20 and 30 min dramatically increased the aldehyde concentrations of the samples, and aldehyde concentrations in these samples showed the highest concentrations among all volatile chemical groups. For ketones, roasting processes induced increased concentrations in a time-dependent manner, regardless of the roasting temperature. The roasted beans (210 ℃) showed dynamic changes in concentrations compared to the roasted beans (150 ℃). In sulfur-containing compounds, the concentrations of these volatiles showed dynamic changes after the roasting processes (210 ℃). Sulfur-containing compounds increased the concentrations in a time-dependent manner (210 ℃ from 20 to 30 min). However, roasting processes (150 ℃) did not show any dynamic changes in concentrations of sulfur-containing compounds. In addition, slight changes in other volatiles (furans, pyrroles, pyrazines, and pyridines) were observed during roasting. The concentration of furans increased after roasting, regardless of the roasting temperature. Pyrroles, pyrazines, and pyridines were only generated during the roasting at 210 ℃, and these volatile concentrations increased in a time-dependent manner.

In the roasting process, aldehydes, ketones, and pyrazines are usually increased in coffee by increasing the roasting time and temperature (Laukaleja & Kruma, 2019). Volatile aldehydes (Strecker aldehydes) are mainly generated in coffee beans via Strecker degradation during roasting and are abundant at medium roasting levels (Gigl et al., 2021, Laukaleja and Kruma, 2019). Various ketones are usually detected in coffee and are responsible for the coffee scent (Yoon et al., 2022). Sulfur-containing compounds usually increase at medium roasting levels and are mainly generated by the thermal degradation of sulfur-containing amino acids (cysteine and methionine) (Caporaso et al., 2018, Gigl et al., 2022, Laukaleja and Kruma, 2019). In this study, the dynamic changes in volatile compounds were shown during the higher temperature (210 ℃) roasting processes. Strecker aldehydes and sulfur-containing compounds were dramatically increased after roasting at 210 ℃ for 20 and 30 min. Thus, these two roasting conditions are likely to be considered the medium roasting level suggested by Laukaleja et al. (Laukaleja & Kruma, 2019).

### Volatile patterns in unroasted and roasted beans via multivariate analysis

Volatile patterns in unroasted and roasted beans (150, 210 ℃) were identified via multivariate analysis (PCA and CA) based on the changes in volatiles using E-nose. The results are shown in Fig. 2B-C. In the PCA biplot, a total of PCs showed 78.74 % variance, including 61.20 % (PC1) and 17.54 % (PC2). Most samples were located on the negative axis of PC1. However, the roasted samples at 210 ℃ for 20 and 30 min were located on the positive axis of PC1, and these samples were influenced by aldehydes and pyrazines. Additionally, a roasted sample at 210 ℃ for 30 min was mainly influenced by sulfur-containing compounds, and the other roasted sample at 210 ℃ for 20 min was mainly influenced by furans. Unlike the results of PC1, most roasted beans were located on the negative axis of PC2, and unroasted and roasted beans at 210 ℃ for 30 min were located on the positive axis of PC2. Accordingly, roasting temperatures were mainly influenced by the PC1 axis, and most of the roasted beans were separated by the PC2 axis compared to the unroasted beans, except for a roasted bean at 210 ℃ for 30 min. In CA results, the samples roasted at 210 ℃ for 20 and 30 min were classified as cluster I, the unroasted bean as cluster II, and the others as cluster Ⅲ. The CA results showed that the roasting temperatures could be discriminated against by the clusters. Roasting temperatures contributed to the discrimination among the samples. In this study, the volatile compound patterns in unroasted and roasted coffee beans were identified using chemometrics (PCA and CA). Recent studies have reported that differences in the volatile patterns of coffee, based on e-nose analysis data, were identified according to species and roasting times via chemometrics (Dong et al., 2017). In this study, the volatile patterns were mainly influenced by the roasting temperature and slightly influenced by the roasting time using chemometrics. In this study, volatile patterns were identified via chemometrics according to roasting temperature and time.

### GM-MS-O

**Fig. 3:**
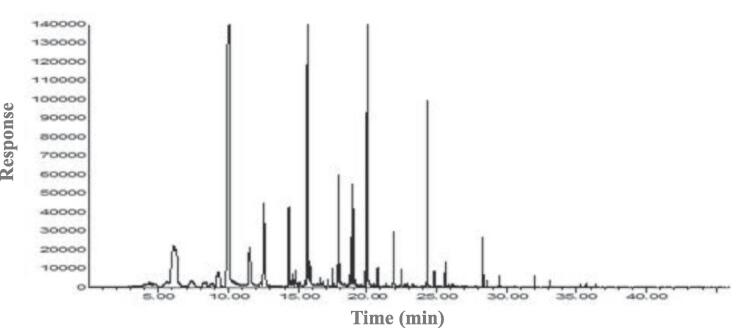

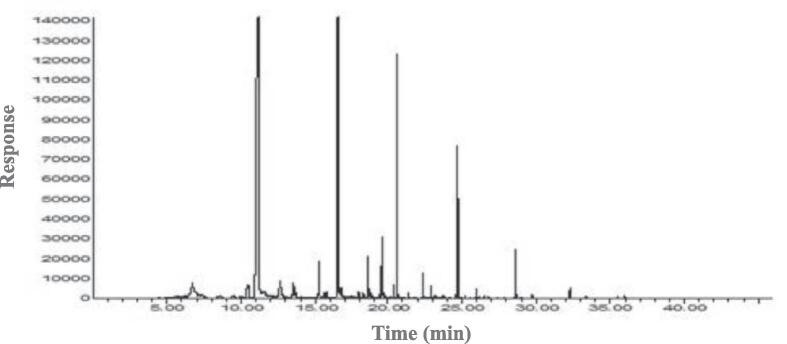

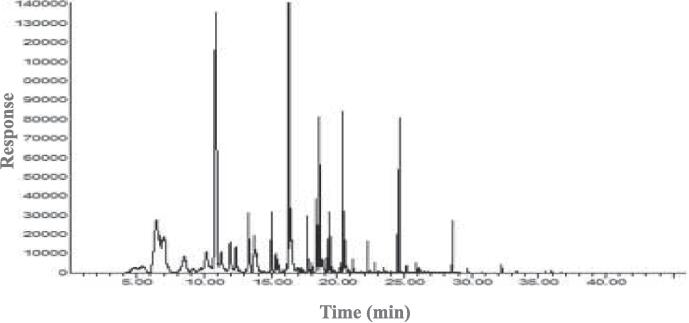

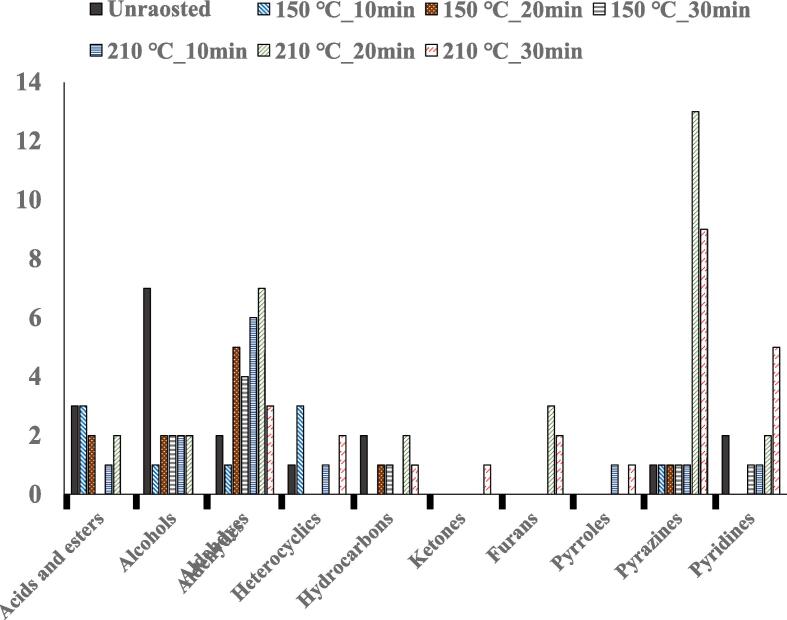

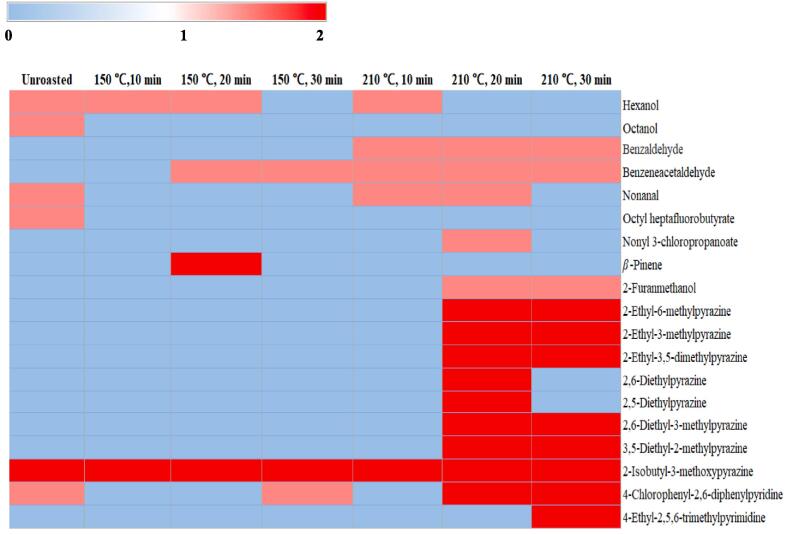

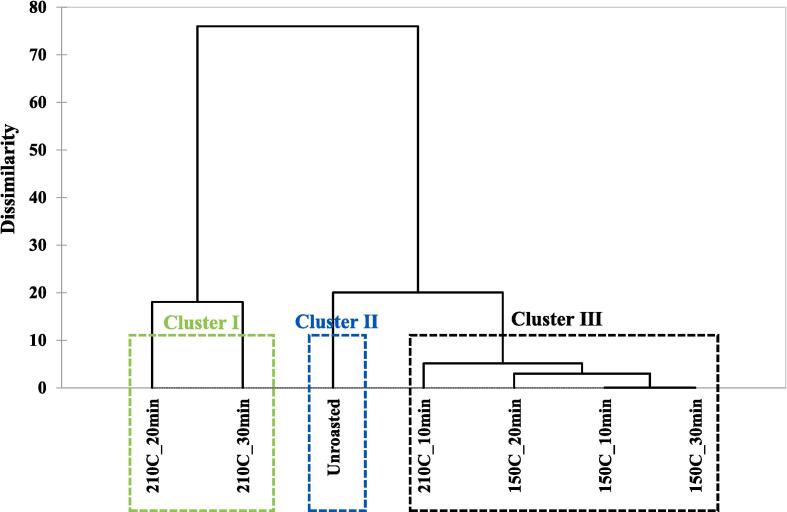
Representative chromatogram of volatile profiles in *Coffea arabica* L. (cv. Yellow Bourbon); unroasted (A), 150 (B), 210 (C), the number of species and the categories of volatile profiles in unroasted and roasted *Coffea arabica* L. (cv. Yellow Bourbon) (D), heat map of the changes in the odor intensity of odor-active compounds for the different roasted coffee beans (E), PCA-biplot for odor-active compound dataset between unroasted- and roasted-coffee samples (F), dendrogram for odor-active compound dataset between unroasted- and roasted-coffee samples (G). (For interpretation of the references to colour in this figure legend, the reader is referred to the web version of this article.)

The volatile compound profiles were analyzed using GC–MS, and the results and chromatograms are shown in Fig. 3A-D. Volatile compounds were identified, including acids, esters, alcohols, aldehydes, heterocyclic hydrocarbons, ketones, furans, pyrroles, pyrazines, and pyridines. The OACs were analyzed using GC–MS-O, and their concentrations and odor intensities are shown in Table 3, Table 4 and Fig. 3E. In unroasted and roasted beans at 150 ℃, a total of 8 OACs were identified, including 2 alcohols, 2 aldehydes, 1 ester, 1 hydrocarbon, 1 pyrazine, and 1 pyridine. A total of 18 OACs were identified at 210 ℃, including 2 alcohols, 3 aldehydes, 2 esters, 1 furan, 8 pyrazines, and 2 pyridines. In unroasted and roasted beans at 150 ℃, octanol, nonanal, and octyl heptafluorobutyrate were only detected in the unroasted bean, and hexanol concentrations did not show any dynamic differences until roasted at 150 ℃ for 20 min. However, a roasted bean at 150 ℃ for 30 min did not contain hexanol. 2-Isobutyl-3-methoxypyrazine was detected in all samples. Benzeneacetaldehyde was only detected during the roasting at 150 ℃ for 20 min and 30 min.

**Table 3:** Qualitative and quantitative analysis of odor-active compounds in coffee extracts according to the roasting processes at 150 ℃.

Compounds	Description	RT^1)^	RI^2)^	Concentration (ug/g)				QM^3)^
		(min)		Unroasted	10 min	20 min	30 min	
**Alcohols(2)**								
Hexanol	Sweet	13.09	963	0.084 ± 0.118	0.083 ± 0.069	0.095 ± 0.101	ND	MS
Octanol	Sweet	17.61	1101	0.021 ± 0.030	ND	ND	ND	MS
**Aldehydes(2)**								
Benzeneacetaldehyde	Bitter, Roast	15.64	1041	ND	ND	0.024 ± 0.002	0.013 ± 0.04	MS
Nonanal	Sweet	18.75	1142	0.079 ± 0.001	ND	ND	ND	MS
**Ester(1)**								
Octyl heptafluorobutyrate	Sweet	17.8	1108	0.017 ± 0.025	ND	ND	ND	MS
**Hydrocarbon(1)**								
*β*-Pinene	Bitter	19.34	1162	ND	ND	0.016 ± 0.001	ND	MS
**Pyrazine(1)**								
2-Isobutyl-3-methoxypyrazine	Bitter, Coffee	20.78	1210	0.025 ± 0.003	0.010 ± 0.014	0.025 ± 0.001	0.005 ± 0.007	MS
**Pyridine(1)**								
4-Chlorophenyl-2,6-diphenylpyridine	Bitter	19.85	1179	0.009 ± 0.013	ND	ND	0.008 ± 0.012	MS

Data are given as mean ± SD values from experiments performed in duplicate.

^1)^ RT: retention time.

^2)^RI: retention index.

^3)^ QM: qualitative method.

**Table 4:** Qualitative and quantitative analysis of odor-active compounds in coffee extracts according to the roasting processes at 210 ℃.

Compounds	Description	RT^1)^	RI^2)^	Concentration (ug/g)				QM^3)^
		(min)		Unroasted	10 min	20 min	30 min	
**Alcohols(2)**								
Hexanol	Sweet	13.09	963	0.084 ± 0.118	0.057 ± 0.081	ND	ND	MS
Octanol	Sweet	17.61	1101	0.021 ± 0.030	ND	ND	ND	MS
**Aldehydes(3)**								
Benzaldehyde	Sweet, Roast	15.64	1041	ND	0.023 ± 0.008	0.064 ± 0.004	0.073 ± 0.070	MS
Benzeneacetaldehyde	Bitter, Roast	17.81	1108	ND	0.052 ± 0.009	0.087 ± 0.061	0.290 ± 0.410	MS
Nonanal	Sweet	18.75	1142	0.079 ± 0.001	0.076 ± 0.007	0.059 ± 0.019	ND	MS
**Esters(2)**								
Octyl heptafluorobutyrate	Sweet	17.80	1108	0.017 ± 0.025	ND	ND	ND	MS
Nonyl 3-chloropropanoate	Sweet	18.31	1126	ND	ND	0.013 ± 0.019	ND	MS
**Furan(1)**								
2-Furanmethanol	Bitter	11.46	913	ND	ND	0.076 ± 0.033	0.051 ± 0.072	MS
**Pyrazines(8)**								
2-Ethyl-6-methylpyrazine	Roast	16.36	1063	ND	ND	0.031 ± 0.014	0.034 ± 0.003	MS
2-Ethyl-3-methylpyrazine	Roast	16.52	1068	ND	ND	0.084 ± 0.001	0.068 ± 0.083	MS
2-Ethyl-3,5-dimethylpyrazine	Bitter, Roast	18.58	1136	ND	ND	0.262 ± 0.046	0.222 ± 0.247	MS
2,6-Diethylpyrazine	Bitter	18.73	1141	ND	ND	0.014 ± 0.020	ND	MS
2,5-Diethylpyrazine	Sweet	18.87	1146	ND	ND	0.012 ± 0.018	ND	MS
2,6-Diethyl-3-methylpyrazine	Coffee	20.61	1204	ND	ND	0.011 ± 0.016	ND	MS
3,5-Diethyl-2-methylpyrazine	Roast, Coffee	20.69	1207	ND	ND	0.034 ± 0.048	0.005 ± 0.007	MS
2-Isobutyl-3-methoxypyrazine	Bitter, Coffee	20.78	1210	0.025 ± 0.003	0.015 ± 0.001	0.017 ± 0.003	0.012 ± 0.017	MS
**Pyridines(2)**								
4-Chlorophenyl-2,6-diphenylpyridine	Bitter	5.33	<800	0.009 ± 0.013	ND	0.009 ± 0.012	0.005 ± 0.007	MS
4-Ethyl-2,5,6-trimethylpyrimidine	Roast	11.39	910	ND	ND	ND	0.057 ± 0.081	MS

Data are given as mean ± SD values from experiments performed in duplicate.

^1)^RT: retention time.

^2)^RI: retention index.

^3)^QM: qualitative method.

In unroasted and roasted beans at 210 ℃, octanol, and octyl heptafluorobutyrate were only detected in unroasted beans, and these OACs were not detected after the roasting processes. Additionally, nonanal, 2-isobutyl-3-methoxypyrazine, and 4-chlorophenyl-2,6-diphenylpyridine, aldehydes (benzaldehyde and benzeneacetaldehyde), and 2-furanmethanol were generated during the roasting processes at 210 ℃. Among the pyridines, 4-Chlorophenyl-2,6-diphenylpyridine concentrations decreased during roasting; however, 4-ethyl-2,5,6-trimethylpyrimidine was detected only after 30 min. Most pyrazines (except 2-isobutyl-3-methoxypyrazine) were generated after 20 min, and the concentrations of these OACs were reduced after roasting for 30 min. Aldehydes showed sweet and roasted odors in the roasted samples. Pyrazines played a key role in odor activation (coffee, roasted, and bitter odors) and intensity during roasting for 20 and 30 min (Fig. 3C).

Among all the OACs, octanol, and octyl heptafluorobutyrate were only found in unroasted beans. Octanol has been reported to contain this compound in unroasted beans, but it was not detected during cooking (Rodríguez-Bernaldo De Quirós, López-Hernández, González-Castro, De la Cruz-García, & Simal-Lozano, 2000). Octyl heptafluorobutyrate was detected only in unroasted beans, and volatile esters produced fruity and floral aromas, which were primarily generated by esterification between acids and alcohols (Zhang et al., 2021). 2-Isobutyl-3-methoxypyrazine was detected in all samples. Accordingly, these three volatiles may be strongly associated with inherent Yellow Bourbon odors. At 150 ℃, roasted beans showed only slight differences in OACs compared to that of unroasted beans. Various aldehydes, furans, pyrazines, and pyridines were generated during roasting. However, the dynamic changes in OACs were shown in higher temperature (210 ℃) roasting processes. Particularly, the generation of aldehydes and pyrazines was shown during the roasting at 210 ℃ for 20 and 30 min, and pyrazine concentrations were also dramatically increased at that condition. Aldehydes are generated during roasting and are closely related to the degradation of unsaturated fatty acids via thermalization (Zhang, Shen, Cao, Duan, & Sun, 2023). Pyrazines are highly related to the roasting process and are mainly found in roasted foods, such as coffee beans, grains, and vegetables (Hong et al., 2022, Yoon et al., 2022). Pyrazines are directly generated via the Maillard reaction between amino acids and reducing sugars during roasting, and the Maillard reaction is not only involved in the generation of pyrazines but also furans and pyridines (Yang et al., 2016, Yoon et al., 2022, Zhang et al., 2023). A previous study reported that pyrazines were highly correlated with roasted and nutty flavors (Zhang et al., 2023), and another study reported a positive correlation between pyrazine generation and roasting time (Yang et al., 2016). Additionally, pyrazines were more likely to be generated at a higher temperature (200 ℃) compared to a lower temperature (140 ℃) (Hong et al., 2022). Consistent with previous research, this study also suggested that pyrazines played an important role in the odor activation and Maillard reaction of roasted coffee beans at 210 ℃ for 20 and 30 min.

### Odor active compound patterns in unroasted and roasted beans via multivariate analysis

OAC patterns in unroasted and roasted beans at 150 and 210 ℃ were identified via multivariate analysis (PCA, CA) based on the changes in OACs using GC-O. The results are shown in Fig. 3F-G. In the PCA biplot, a total of PCs showed 71.68 % variance, including 53.65 % (PC1) and 18.03 % (PC2). Most of the samples were located on the negative axis of PC1, however, other roasted samples at 210 ℃ for 20 and 30 min were located on the positive axis of PC1. In the PC2 results, an unroasted bean was located on the upper side of all beans, and the roasted beans shifted downward compared with the unroasted beans. Particularly, all roasted beans at 150 ℃ and the roasted beans at 210 ℃ for 30 min were located on the negative axis of PC2. However, roasted beans at 210 ℃ for 10 and 20 min were located on the positive axis of PC2. An unroasted bean was influenced by octanol and octyl heptafluorobutyrate, and a roasted bean at 210 ℃ for 10 min was influenced by hexanol. All roasted beans at 150 ℃ were influenced by 2-Isobutyl-3-methoxypyrazine. The roasted beans at 210 ℃ for 20 and 30 min were mainly influenced by pyrazines. A roasted bean at 210 ℃ for 20 min was mainly influenced by three pyrazines (2,6-dimethyl-3-methylpyrazine, 2,6-dimehtylpyrazine, 2-isobutyl-3-methoxypyrazine), nonyl 3-chloropropanoate, and 4-chlorophenyl-2,6-diphenylpyridine. However, roasted bean at 210 ℃ for 30 min was mainly influenced by five pyrazines (3,5-diethyl-2-methylpyrazine, 2-ethyl-3-methylpyrazine, 2-ethyl-6-methylpyrazine, 2,5-dimethylpyrazine, 2-ethyl-3,5-dimethylpyrazine) and 2-furanmethanol. In CA results, the roasted beans at 210 ℃ for 20 and 30 min were classified as cluster Ⅰ, the unroasted bean was classified as cluster Ⅱ and other roasted beans were classified as cluster Ⅲ. Among all clusters, Cluster I and the other clusters showed relatively high dissimilarity with many pyrazines (PCA results). Previous Arabica coffee aroma research using CA reported that similar structures of pyrazines showed relatively higher similarity than other volatiles with similar structures (Caporaso et al., 2018). Similarly, our results were also consistent with previous research, and this study also identified that pyrazines influenced similar roasting conditions (210 ℃ for 20 and 30 min). Additionally, pyrazines were identified as the main variable among all the OACs during the roasting process.

## Conclusions

In this study, coffee beans were roasted at different temperatures and times, and their physicochemical, taste, and volatile profiles were measured using HPLC, electronic sensors, and GC–MS-O. The extension of roasting time increased the TFC of roasted coffee beans, and the caffeine concentrations tended to increase during roasting. Chlorogenic acid increased during the roasting at 150 ℃, however, it decreased during the roasting at 210 ℃. The DPPH radical scavenging activity did not show any significant differences during roasting. In E-tongue analysis, SRS and UMS values decreased during the roasting at 150 ℃, and no dynamic changes occurred in STS, BRS, or SWS. However, BRS and SWS values increased, and STS and SRS values decreased during the roasting at 210 ℃. No dynamic changes were observed in UMS during the roasting at 210 ℃ compared to that at 150 ℃. In E-nose analysis, aldehydes, ketones, and sulfur-containing compounds dramatically increased at 210 ℃ for 20 and 30 min during the roasting. In GC-O analysis, pyrazines were mainly generated during the roasting at 210 ℃ for 20 and 30 min, and pyrazines showed the highest concentrations among all OACs. Additionally, pyrazines exhibit coffee, bitter, and roasted odors. In the multivariate analysis the results of the e-tongue multivariate analysis showed the separation of beans according to the temperature. The results of the E-nose and GC-O multivariate analyses showed the separation of beans according to temperature and time. Accordingly, the impacts of taste patterns were mainly influenced by roasting temperature, while the impacts of volatile (E-nose) and OACs (GC-O) patterns were mainly influenced by roasting temperature and time. However, our research had a few limitations, and further studies should investigate the relationship between the human senses (human sensory test) and human-mimicking senses (electronic sensors).

## CRediT authorship contribution statement

**Seong Jun Hong:** Methodology, Formal analysis. **Chang Guk Boo:** Conceptualization, Formal analysis, Writing – original draft. **Sojeong Yoon:** Methodology, Formal analysis. **Hyangyeon Jeong:** Methodology, Formal analysis. **Seong Min Jo:** Methodology, Formal analysis. **Moon Yeon Youn:** Supervision, Writing – review & editing. **Jae Kyeom Kim:** Conceptualization, Writing – review & editing. **Young Jun Kim:** Conceptualization, Writing – review & editing. **Eui-Cheol Shin:** Writing – review & editing, Supervision, Methodology, Conceptualization.

## Declaration of competing interest

The authors declare that they have no known competing financial interests or personal relationships that could have appeared to influence the work reported in this paper.

## Data Availability

Data will be made available on request.
